# Assessment of Environmental Sustainability and Corporate Social Responsibility Reporting by Large Health Care Organizations

**DOI:** 10.1001/jamanetworkopen.2018.0975

**Published:** 2018-08-03

**Authors:** Emily Senay, Philip J. Landrigan

**Affiliations:** 1 World Trade Center Health Program Clinical Center of Excellence, Selikoff Centers for Occupational Health, Division of Preventive Medicine, Department of Environmental Medicine and Public Health, Icahn School of Medicine at Mount Sinai, New York, New York; 2 Arnhold Institute for Global Health, Icahn School of Medicine at Mount Sinai, New York, New York

## Abstract

**Importance:**

Sustainability practices by large corporations are increasingly important to reduce the environmental and social impacts of the business enterprise. The health care industry represents 18% of the US economy, employs more people than any other economic sector, and is responsible for 10% of US carbon emissions. The engagement of large health care delivery organizations in reporting sustainability efforts has not been previously assessed.

**Objective:**

To evaluate sustainability reporting by large US health care delivery organizations compared with reporting trends in all other economic sectors.

**Design, Setting, and Participants:**

Cohort study of 49 large health care organizations (HCOs) by inclusion on one of the following lists in 2015 or 2016: Fortune 500, S&P (Standard & Poor) 500, Forbes 100 Largest Charities, Becker’s Hospital Review of largest HCOs, and 24/7 Wall St’s largest state employers. Each HCO was analyzed for sustainability reporting by reviewing the main corporate website, Google search, and search of publicly available databases. The percentage of sustainability reporting by HCOs on each list was compared with the percentage of sustainability reporting by all corporations on each list as obtained from public reports and publicly available databases. Data analysis was conducted in January 2018.

**Main Outcomes and Measures:**

The percentages of large health care corporations and other corporations publicly reporting sustainability information.

**Results:**

Forty-nine large for-profit and nonprofit US HCOs were analyzed (10 appeared on >1 list but were analyzed only once) appearing on the Fortune 500 (8 [16%]), S&P 500 (3 [6%]), Forbes 100 Largest Charities (8 [16%]), largest state employers (14 [29%]), largest for-profit HCOs (11 [22%]), and largest nonprofit HCOs (17 [35%]) by facilities owned for sustainability reporting. Among them, 4 of 8 (50%) on the Fortune 500, 1 of 3 (33%) on the S&P 500, and 6 of all 49 health care corporations (12%) published a sustainability report compared with 389 of 500 (78%) on the Fortune 500 and 410 of 500 (82%) on the S&P 500 reporting by all economic sectors.

**Conclusions and Relevance:**

The health care delivery sector lags behind other US economic sectors in sustainability reporting. Publicly reporting sustainability activities would provide HCOs with an incentive to quantify and reduce their environmental impacts, lower costs, and protect human health.

## Key Points

**Question:**

Do large health care organizations participate in the business trend to report on sustainability activities?

**Findings:**

In this cohort study of 49 large US health care organizations appearing on 2015 or 2016 lists of the largest US corporations (Fortune 500, S&P 500, Forbes 100 Largest Charities, Largest State Employers, and largest health care systems by facilities owned), 50% of Fortune 500, 33% of S&P 500, and 12% of all health care corporations published a sustainability report compared with 78% of Fortune 500 and 82% of S&P 500 corporations.

**Meaning:**

Sustainability reporting would provide health care organizations with an incentive to quantify and reduce their environmental impact, reduce operating costs, and enhance protection of human health.

## Teaser

This study characterizes environmental sustainability and corporate social responsibility (CSR) reporting by US health care organizations (HCOs) named by Fortune 500, Forbes, and other financial sources as the largest US HCOs in 2015 or 2016 and compares CSR reporting trends among those HCOs with reporting in other economic sectors.

## Introduction

Over the past 2 decades many large corporations have begun to acknowledge that they must hold themselves responsible and take measures to reduce the environmental degradation, pollution, climate change, and social disruption that results from their activities. This represents a major transformation in the business landscape that challenges the traditional model that corporations are responsible only to their shareholders without regard to the social and environmental consequences of their actions. At the same time, investors and other stakeholders, such as governments and consumers, are increasingly demanding transparency from corporate leadership about the social and environmental impacts of business operations.

To meet these demands many companies now publish annual reports detailing their efforts to measure, manage, and mitigate their environmental and social impacts. Commonly referred to in the business world as corporate social responsibility (CSR) reporting, these efforts are also known as sustainability reporting, nonfinancial reporting, integrated reporting, corporate citizenship reporting, triple bottom line reporting, and environmental, social, and governance reporting. Historically, these reports began as a platform to highlight corporate philanthropy but have evolved to include corporate performance on environmental measures (waste, water, and pollution) and on social impacts such as workforce well-being, diversity and equality practices, labor and management relations, human rights, and effects on local communities throughout the supply chain. For this article we refer to sustainability reporting to reference activities primarily in the environmental sphere and CSR to reflect a formal published report and/or activities that broaden efforts to include some degree of social impacts. Corporate social responsibility reports, which are currently voluntary in the United States and distinct from regulatory reporting or required US Securities and Exchange Commission corporate financial filings, have become the conventional format for companies to communicate performance on environmental, social, and governance issues and are now published by a majority of the largest publicly traded companies as well as many private and nonprofit companies. In 2016, 82% of S&P (Standard & Poor) 500 publicly traded companies published a CSR report compared with just 20% reported in 2011.^1^ In 2015, 90% of the world’s 250 largest companies published a CSR report.^2^ The Centre for Sustainability and Excellence, a US-based sustainability management consulting firm, analyzed 550 North American companies and found 79% published a CSR report in 2015 and 2016.^3^ In addition to preparing CSR reports, many large corporations signal their commitment to environmental sustainability by disclosing their carbon emissions to third-party accounting groups. In 2017, 70% of S&P 500 companies voluntarily reported their carbon emissions to the Carbon Disclosure Project, a UK-based nongovernmental organization operating a global disclosure system to help companies, cities, and governments report on and manage their carbon emissions; to date, more than 6000 companies from around the world have disclosed their carbon emissions.^4^

More recently, pressure to report sustainability and corporate responsibility activities intensified in 2017 when Vanguard, the largest provider of mutual funds, with $4.5 trillion in assets, notified publicly traded companies seeking support that it wants full disclosure of all sustainability risks. In early 2018, BlackRock, the world’s largest investor with $6.3 trillion under management, notified chief executive officers that it will no longer seek investments in companies that do not demonstrate CSR activities.

To standardize reporting across all economic sectors, a number of reporting frameworks have been developed and are now used by a majority of companies. One of the most common frameworks is promulgated by the Global Reporting Initiative (GRI), an independent standards organization that maintains a searchable database of CSR reports of companies from around the world. Despite increasing stakeholder pressure and the availability of frameworks, the sophistication, depth, transparency, and quality of sustainability and CSR reporting is still evolving. As such, the act of sharing sustainability information via websites or publishing a CSR report does not necessarily verify that a corporation is maximizing corporate citizenship efforts.

The health care industry is one of the largest and fastest growing economic sectors in the United States, representing 17.7% of the GDP in 2015 with projections to reach 20% by 2025.^5^ In 2017, for the first time, health care surpassed retail and manufacturing to become the largest source of jobs in the United States.^6^ Spurred by the passage of the Affordable Care Act and the move to value-based reimbursement models, mergers and acquisitions among health care organizations (HCOs) increased by 55% between 2010 and 2016,^7^ resulting in larger corporate entities and highly concentrated hospital markets in 90% of metropolitan areas.^8^

Health care organizations are now among the largest corporations in the United States and generate large revenues. For example, Hospital Corporation of America Holdings, the largest for-profit health system in the United States, had more than $44 billion in revenue in 2016 and ranked 63rd on the Fortune 500.^9^ Kaiser Permanente, the nation’s largest nonprofit health system, generated more than $64 billion in revenue in 2016.^10^ If the organization was eligible for the Fortune 500 list, it would rank 39th, ahead of MetLife, Pepsi, and Disney.

The US health care delivery industry consumes vast resources, the majority of which become waste—nearly 7000 tons of hospital waste is created daily.^11^ The health care delivery sector is responsible for 10% of all greenhouse gas emissions, 12% of acid rain, 10% of smog formation, and 9% of criteria air pollutants (ground-level ozone, particulate matter, carbon monoxide, lead, sulfur dioxide, and nitrogen dioxide), which leads to indirect health burdens commensurate with the 44 000 to 98 000 hospital deaths each year from preventable medical errors.^12^

Corporate social responsibility activities have been shown to provide a positive return on investment; enhance employee recruitment, retention, productivity, and well-being; and create positive consumer sentiment.^13^ There is natural synergy between the mission of health care delivery, sustainability, and CSR activities. All seek to improve human well-being, the health care enterprise directly through the provision of medical care, sustainability by improving the environment, and CSR by including efforts to improve the social welfare of employees, consumers, and communities. The degree to which large health care corporations participate in the business trends to report on sustainability and/or CSR activities by publishing reports or by providing information via corporate websites is evaluated in this article.

## Methods

Health care delivery companies were defined as companies that own and operate facilities providing direct patient care. Large health care delivery companies were defined by their inclusion on one of the following lists: 2016 Fortune 500,^9^ S&P 500,^14^ Forbes 100 Largest Charities,^15^ 2015 largest for-profit^16^ and nonprofit^17^ health care systems by number of facilities complied by Becker’s Hospital Review, and June 2016 largest employer in every state compiled by 24/7 Wall St.^18^ This study follows the Strengthening the Reporting of Observational Studies in Epidemiology (STROBE) reporting guideline.

The GRI database^19^ was searched for a published CSR report for each of the large health care delivery corporations. Many large corporations with robust sustainability or CSR programs prominently feature their efforts on their main corporate website. Therefore, sustainability and/or CSR information included on the main webpage was considered a proxy for importance given these activities by corporate leadership. Each large health care corporation was evaluated through a search of the main corporate website landing page or drop-down menu on the landing page and through Google search with corporate name and at least 1 of the following terms: sustainability, corporate responsibility, social responsibility, citizenship, or environment. If no main website information was found and/or no CSR report was identified via the main website or GRI database, a Google search was performed to determine if corporate materials on basic sustainability activities (eg, waste, energy, recycling, reprocessing, and carbon dioxide [CO_2_] emissions) were available elsewhere. This was used as a proxy to signal some level of corporate awareness of basic sustainability activities but reflecting less of a corporate commitment than main website mention or the publication of a CSR report. A sustainability metric was considered to be mentioned if it included a quantity (pounds, gallons, British thermal units, etc), whether or not it was benchmarked or tracked over time. Mention of CO_2_ was considered to be “yes” if emissions were noted as total emissions for the institution or goals in reduction were described numerically (without total emissions noted or report to the Carbon Disclosure Project). Mention of at least 1 sustainability metric was considered to be a proxy to help distinguish between a narrative in corporate materials that conveys support for sustainability concepts vs some evidence of actual or potential measurement and management of waste generation and energy use.

We individually analyzed in detail only the health care corporations on each list of large US corporations, since data for total CSR reporting was readily available via published reports or through search of publicly available databases. The total percentage of CSR reporting by all corporations on the 2016 S&P 500 was compiled and publicly reported by the Governance & Accountability Institute, Inc.^1^ Reporting of CO_2_ emissions by all corporations on the S&P 500 was compiled and published by the Carbon Disclosure Project.^20^ The total percentage of sustainability reporting and CO_2_ emissions disclosure by corporations on the 2016 Fortune 500 and 2016 Forbes 100 Largest Charities was compiled by CSRHub, the largest database of corporate social responsibility, environmental, and governance ratings and information worldwide.^21^ Reporting of CSR in these databases included GRI as well as other reporting formats. For each list, percentages of health care corporations reporting sustainability activities ascertained by our analysis were compared with the total percentage of sustainability reporting for all corporations on each list as compiled by the mentioned firms.

## Results

A total of 49 health care corporations were analyzed (10 appeared on >1 list but were analyzed only once). In 2016, 8 health care corporations appeared on the Fortune 500, 3 on the S&P 500, and 8 on the Forbes 100 Largest Charities. In 2016, HCOs were the top employers in 14 states and in 2015 a total of 28 for-profit and nonprofit health systems were listed as the largest by number of facilities owned and operated. Because of ties in the number of facilities owned and operated, 11 top 10 largest for-profit HCOs and 17 top 10 largest nonprofit HCOs were included (Table).

**Table.** Demographics of HCOs Analyzed

List	No. of HCOs							
	Total	For-Profit	Nonprofit	Northeast	Southeast	Midwest	Southwest	West
Fortune 500	8	8	0	1	4	0	1	2
S&P 500	3	3	0	1	1	0	0	1
Forbes 100 Largest Charities	8	0	8	4	2	2	0	0
10 Largest for-profit HCOs	11	11	0	2	6	1	0	2
10 Largest nonprofit HCOs	17	0	17	2	3	2	3	7
Largest state employers	14	7	7	7	0	4	0	3

Abbreviations: HCO, health care organization; S&P, Standard & Poor.

Of the 49 HCOs analyzed, 6 (12%) published a CSR report as determined by a search of the GRI database: 4 of 8 (50%) on the Fortune 500; 1 of 3 (33%) on the S&P 500; 1 of 8 (13%) on the Forbes 100 Largest Charities; 2 of 11 (18%) of the largest for-profit HCOs; and 1 of 17 (6%) of the largest nonprofit HCOs by number of facilities owned and operated (some companies appeared on >1 list). No health care corporation on the largest state employer list published a CSR report. Mention of sustainability terms were found on the main corporate website for health care corporations as follows: 2 of 8 (25%) on the Fortune 500; 1 of 3 (33%) on the S&P 500; 5 of 14 (36%) of the largest state employers; 1 of 11 (9%) of the largest for-profit HCOs; and 5 of 17 (29%) of the largest nonprofit HCOs by number of facilities owned and operated. Mention of CO_2_ emissions by health care corporations were as follows: 1 of 8 (13%) on the Fortune 500; 2 of 14 (14%) of the health care top state employers; 1 of the 11 (9%) of the largest for-profit HCOs; and 5 of 17 (29%) of the largest nonprofit HCOs by facilities owned and operated. No S&P 500 health care company mentioned CO_2_ emissions.

Our findings for health care corporations on the Fortune 500, S&P 500, and largest state employers were compared with the data for all corporations available from published reports and publicly available databases. Among all corporations, 389 of 500 (78%) on the Fortune 500,^21^ 410 of 500 (82%) on the S&P 500,^1^ and 24 of 36 (67%) of non–health care top state employers published a CSR report, and 261 of 500 (52%) on the Fortune 500,^21^ 385 of 500 (77%) on the S&P 500,^4^ and 31 of 36 (86%) of non–health care top state employers reported CO_2_ emissions (Figure). Of the 12 top state employer nonprofit state university systems, 8 (66%) mentioned CO_2 _emissions.

**Figure.**
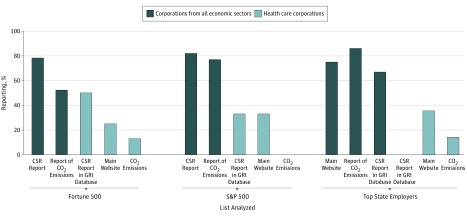
Sustainability Reporting by Health Care Sector Compared With All Sectors Sources are the 2016 Fortune 500, S&P 500, and Forbes 100 Charities; June 2016 largest employer in every state was compiled by 24/7 Wall St. Corporate social responsibility (CSR) data for companies on the 2016 S&P 500 list were obtained from the Governance & Accountability Institute, Inc and from the Carbon Disclosure Project. Data for companies on the 2016 Fortune 500 were compiled by CSRHub. Data from the the Global Reporting Initiative (GRI) database and corporate websites of all health care corporations were from the authors’ analysis. CO_2_ indicates carbon dioxide; S&P, Standard & Poor.

If no CSR report was found and sustainability activities were not mentioned on the main corporate website, a Google search was performed to determine if other health care corporate material mentioned sustainability terms. Results were as follows: 5 of 8 (63%) on the Fortune 500; 2 of 3 (66%) on the S&P 500; 8 of 14 (64%) of the largest state employers; 4 of 11 (36%) of the top state for-profit HCOs; and 8 of 17 (47%) of the top state nonprofit HCOs. Of the 12 top state employer nonprofit state university systems, 11 (92%) mentioned sustainability via Google search. Mention of at least 1 sustainability metric was identified for health care corporations as follows: 5 of 8 (63%) on the Fortune 500; 2 of 3 (66%) on the S&P 500; 4 of 14 (14%) of the largest state employers; 4 of 11 (36%) of the largest for-profit HCOs; and 5 of 17 (29%) of the largest nonprofit HCOs by number of facilities owned and operated.

Search of the CSRHub database for reporting by Forbes 100 Largest Charities revealed few published CSR reports. Nevertheless, we analyzed all 8 health care corporations on the Forbes 100 Largest Charities list and found the following: 1 of 8 (13%) published a CSR report found in the GRI database; 1 of 8 (13%) mentioned a sustainability on the main website; 5 of 8 (63%) mentioned sustainability elsewhere via Google search; 3 of 8 (38%) reported at least 1 sustainability metric; and 1 of 8 (13%) mentioned CO_2_ emissions.

## Discussion

To our knowledge, this is the first assessment of sustainability reporting by large corporations in the health care delivery sector. Our analysis finds the health care delivery sector lags far behind other economic sectors in communicating sustainability activities. In 2016, 389 of 500 (78%) of Fortune 500 companies and 410 of 500 (82%) of S&P 500 companies reported CSR activities compared with 4 of 8 (50%) Fortune 500, 1 of 3 (33%) S&P 500, and 6 of 49 (12%) of all health care corporations appearing on any list. If mention of sustainability terms on the main corporate website is a proxy for corporate commitment to these activities, then health care corporations lag; only 2 of 8 (25%) of Fortune 500 companies; 1 of 3 (33%) of S&P 500 companies, 1 of 8 (13%) of Forbes 100 Largest Charities, and 5 of 14 (36%) of top state employers mentioned sustainability or CSR terms on their main websites. Furthermore, 77% of all S&P 500 and 52% of all Fortune 500 companies disclosed carbon emission information to the Carbon Disclosure Project, but only 1 of 8 (13%) of Fortune 500 health care companies, 1 of 8 (13%) of Forbes 100 Largest Charities, 2 of 14 (14%) of the largest state employer health care systems, and no S&P 500 health care companies mentioned carbon emissions in corporate material.

While overall performance in sustainability reporting is poor compared with other economic sectors, there are indications that these activities have some importance to health care corporations. A greater number of health care corporations were found to mention basic sustainability information when searched via Google: 2 of 3 (66%) of S&P 500, 5 of 8 (63%) of Fortune 500, and 8 of 14 (64%) of the largest state employer HCOs. Most also included at least 1 metric, which provides some evidence of commitment to sustainability; however, without publishing a CSR report or including mention of these efforts on the main website, it is difficult to determine to what degree HCOs truly value and pursue these activities.

Perhaps the starkest contrast in reporting is seen in the top state employers. In 2015 Walmart was the largest employer in 19 states; Boeing, Intel, MGM Grand, Hannaford Supermarkets, and Wakefern Foods were the largest in 1 state each. All of these large corporations have robust sustainability programs, include CSR on their websites, and publish CSR reports, and all but 1 reported CO_2 _emissions. By contrast, only 5 of 14 (36%) of top state employer HCOs included sustainability information on their main website, only 1 mentioned CO_2_ emissions, and none had CSR reports searchable via GRI.

It could be argued that private and charitable corporations are exempt from sustainability or more robust CSR activities because they do not face shareholder pressure. However, many large private companies do participate in some form of CSR. For example, of the 5 largest privately held corporations in the United States—Cargill, Koch, Mars, PricewaterhouseCoopers, and Bechtel—only Koch Industries does not publish any form of sustainability information. Furthermore, nonprofit state university systems—the top employers in 12 states—are more likely to mention sustainability via a Google search (11 of 12 [92%]) and mention CO_2_ emissions (8 of 12 [66%]) than top state employer HCOs. It is noteworthy that the largest nonprofit HCOs by number of facilities owned are in fact more likely than the largest for-profit HCOs to address sustainability and report CO_2 _emissions (5 of 17 [29%] vs 1 of 11 [9%]) for both measures.

Efforts of CSR can yield a large positive return on investment. An examination of the potential cost savings that could be realized from application of best practices in energy and waste management for the US health care system estimated a return on investment of $5.4 billion over 5 years and $15 billion over 10 years.^22^ Reduction of pollution and greenhouse gas emissions would also provide a cobenefit for human health by reducing disease burden and medical costs, all of which directly support the mission of health care.

### Limitations

Limitations of this study include the possibility that large health care corporations are actively involved in sustainability or CSR activities but do not communicate them publicly via websites or published reports. Additionally, we searched only the GRI database for a published report and no other reporting frameworks, therefore possibly underestimating reporting by health care corporations. Additionally, we directly assessed only health care corporations and not all corporations, relying instead on published reports and databases for overall reporting. Therefore, we may have overestimated or underestimated reporting by all corporations on each list. In the business world, many companies with robust CSR programs feature their reports prominently on the main corporate website. In our analysis we made the assumption that if it is not on the main website, it is of lower priority to corporate leadership. It was our judgment that inclusion on the main corporate website is a better indicator of the importance given to sustainability or CSR activities by corporate leadership whether or not a formal CSR report was identified; however, we did not test this assumption directly. Furthermore, we did not directly survey any corporation to ascertain attitudes, actual sustainability, and CSR activities as opposed to what they are reporting. We may give a false impression of inactivity or lack of corporate commitment to sustainability and CSR on the part of the health care delivery sector. In addition, the rapid pace of corporate mergers in the health care delivery sector, spurred by the transition to population-based medicine, may mean corporate leadership has not had time to gather and publish relevant sustainability or CSR information. It is also important to note that we focused primarily on environmental metrics for our analysis and not on broader social impacts (eg, employee well-being and human rights) that are also key components of CSR. Therefore, it is possible that we have underestimated efforts on social measures by health care corporations. Additionally, we looked only at large economic entities and not midsize or small corporations and our analysis may not accurately reflect the totality of efforts by the health care delivery sector since many smaller entities may be engaged in and reporting on sustainability and/or CSR activities. Finally, this report is limited by the fact it is a descriptive assessment with no statistical analysis.

## Conclusions

This study did not explore why health care corporations have lagged behind corporations in other economic sectors in adopting sustainability reporting. Further studies should evaluate to what degree this is associated with challenges such as slim operating margins, regulatory burdens, political uncertainty, evolving payment models, lack of familiarity with CSR, misperceptions about cost-effectiveness, and a sentiment that charity status and the already substantial societal benefit of providing medical care exempt HCOs from CSR. Our analysis provides no information about the actual quality, depth, and impact of sustainability and/or CSR activities in any economic sector.

Despite the overall paucity of sustainability reporting in the health care sector, efforts to improve the footprint of the health care industry have been under way for years championed by programs such as the American Hospital Association’s Sustainability Roadmap for Hospitals, Healthcare Without Harm, and Practice Greenhealth. Numerous case studies have been published of health facilities that have reduced costs while improving their environmental footprint. Many health systems are innovating ways to build sustainable, LEED (Leadership in Energy and Environmental Design)–certified facilities (a rating system for environmentally responsible construction practices)^23^ and to incorporate wellness design aimed at improving recovery time through natural light, views, and ventilation. New York City hospitals have signed on to an ambitious pledge from the mayor to reduce carbon emissions by at least 40% by 2030. A few early CSR adopters such as Kaiser Permanente and Cleveland Clinic have kept pace with the business world, and Cleveland Clinic publishes a formal CSR report annually.

Despite these exemplars, the business world has substantially surpassed the health care delivery industry in responding to the demand for greater accountability for the environmental and social impacts of the enterprise by creating frameworks and metrics for measuring and reporting on sustainability and social responsibility. It is a business maxim that “you can’t manage what you don’t measure” and these readily available frameworks would help align the business of health care with its mission and create uniformity in reporting, providing greater accountability and better information for all stakeholders. The health care delivery sector has the opportunity to lead in sustainability and CSR—no other industry has a mission that approximates so closely the ultimate goal of all CSR activities—the protection of the environmental and social systems that protect and enhance human health and well-being. Furthermore, greater participation by the health care sector in sustainability efforts is consistent with the intent of the United Nations (UN) Global Compact and will advance attainment of the UN Sustainable Development Goals. The UN Global Compact, also called the world’s largest corporate sustainability initiative, is an effort to enlist corporations worldwide to align their business strategies and operations with universal principles of human rights, labor, environment, and anticorruption. To date, more than 9500 companies from 160 countries in the developed and developing world are signatories to the UN Global Compact. A strong argument could be made that health care delivery corporations should align their operations with these goals. Corporate social responsibility continues to evolve but it is not likely to be a passing fad. It is safe to say that no economic sector will be exempted from the expectation for good corporate citizenship; in this regard, health care corporations are part of the problem but critical to the solution.
